# Supplementary material for: Interpersonal discrimination and depressive symptoms among older Black and African American adults
Source: PLoS One. 2024 Jun 6;19(6):e0304168. doi: 10.1371/journal.pone.0304168 (PMC11156267; doi:10.1371/journal.pone.0304168)
Supplement: Attachment — Submitted filename: Response to reviewers.docx [file pone.0304168.s002.docx]

Thank you for you

*1 - First of all it's important the paper follows the Plos One Submission Guidelines (Especially Reference style, Headings, Page and line numbers).*

Thank you for the feedback. We have updated the formatting reflect that of what is required per PLoS One submission guidelines
 *2 - Introduction - For greater clarity, it would be interesting to eliminate the subsections.*
This was very helpful. We have removed the subheadings from the introduction as suggested.

3 - *Methods / Participants - “Participants were adults aged 50 and older who identified as Black or African American and who reported they had either used alcohol, cannabis, or prescription opioids or sedative tranquilizers at least once in the past month”. - Why were people selected who reported they had either used alcohol, cannabis, prescription opioids, or sedative tranquilizers at least once in the past month? It would be great to specify.*

Thank you for this feedback. We have clarified in the manuscript that the participants were a part of a study depression and discrimination as risk factors for alcohol, cannabis, and prescription opioid or sedative-tranquilizers use.

*4 - Methods / Measures / Vigilant coping - Are there other recent studies that used the Heightened Vigilance Scale (HSV)?*

*This was a great question! We have added information about other uses of this measure more recently with citations.

5 - Methods / Measures / Covariates - Other covariates were tested in the model? Income? Marital Status? Employment Status?*
We appreciate pointing out the choice of covariates. We clarified that covariates were chosen based on preliminary bivariate correlations of participant characteristic variables including demographics, socioeconomic status, health characteristics, and key variables of interest in addition to previous literature. We found that age, gender, and psychiatric diagnosis were both significantly associated with depression, interpersonal discrimination, and heightened vigilance and consistent covariates in literature.

6 - Methods / Analytic strategy - It would be important to specify the model’s adjustments, residual deviance, and which function was used.

We appreciate this feedback. We have clarified that the models were adjusted for by covariates in the adjusted included the unadjusted models with the adjusted models. We also clarified that the models were general multiple linear regression models and not generalized linear models and were more precise in our description. Since we conducted general multiple linear regressions we did not add residual deviances and a function.

*7 - Results - It would be highly important to present results minutely. Please, present the crude and adjusted model. Describing the power of the associations found, not only the p-value.*

Thank you for this suggestion. We have included the unadjusted models with the adjusted models and have added correlation coefficients with the p-values.

*8 - Conclusions - It would be great to present the conclusions in only one section.*

Thank for you this feedback. We have presented the conclusions in one section and removed subheadings.
